# Erbin Confers Neuroprotection against Cerebral Ischemia–Reperfusion Injury in Mice via MAPK Pathway Inhibition

**DOI:** 10.1523/ENEURO.0089-25.2026

**Published:** 2026-05-12

**Authors:** Danyang Meng, Aiming Gu, Beibei Liu, Junjie Xu, Man Luo, Huirong Yan, Zhemeng Chen, Juan Qu, Jin Hu

**Affiliations:** ^1^Department of Neurology, Affiliated Hospital of Jiaxing University, Jiaxing 314000, China; ^2^Jiaxing Institute of Arteriosclerotic Diseases, Affiliated Hospital of Jiaxing University, Jiaxing 314000, China; ^3^Department of Neurology, Jiaxing Hospital of Traditional Chinese Medicine, Jiaxing 314000, China; ^4^Department of Central Laboratory, Affiliated Hospital of Jiaxing University, Jiaxing 314000, China

**Keywords:** Erbin, MAPK, MCAO, p38, stroke

## Abstract

Ischemic stroke, a leading cause of neurological morbidity, is characterized by extensive neuronal injury and a robust inflammatory response. Erbin, a scaffold protein involved in multiple cellular signaling pathways, regulates neuroinflammation and may confer neuroprotection against ischemia–reperfusion (I/R) injury. A mouse model of middle cerebral artery occlusion was utilized to evaluate the neuroprotective role of Erbin. Male mice were allocated into groups receiving either a lentiviral (LV) control vector or LV-mediated Erbin overexpression, followed by I/R injury induction. Neurological function, infarct volume, and expression levels of inflammatory cytokines and mitogen-activated protein kinase (MAPK) signaling proteins were analyzed. Overexpression of Erbin via LV transduction significantly reduced cerebral infarct volume and mitigated neurological impairments post-I/R injury. Furthermore, Erbin overexpression suppressed the phosphorylation of p38 and extracellular signal-regulated kinase in HT22 neuronal cells, indicating attenuation of MAPK pathway activation. Notably, Erbin overexpression modulated the inflammatory response elicited by I/R injury, leading to a reduction in proinflammatory cytokine levels.

## Graphical abstract

**Figure:**
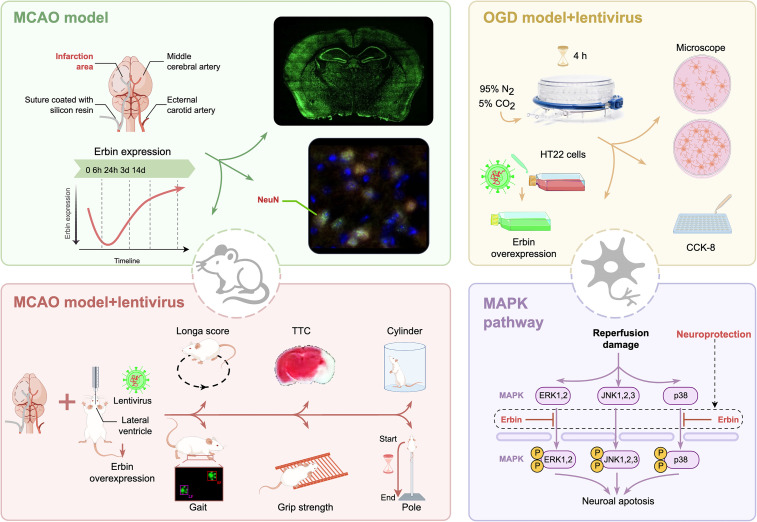
Visual Abstract

## Significance Statement

This study reveals that Erbin overexpression protects against cerebral ischemia–reperfusion injury by inhibiting mitogen-activated protein kinase pathway activation, specifically by inhibiting the phosphorylation of ERK and p38. Erbin mitigates infarct volume, improves neurological function, and suppresses inflammatory cytokines in both in vivo and in vitro models. These findings highlight Erbin as a potential therapeutic target for ischemic stroke, offering a novel strategy to combat neuroinflammation and neuronal damage.

## Introduction

Stroke is a complex neurological disorder resulting from focal cerebral injury due to vascular pathology (Sacco et al., 2013; Feigin et al., 2018; GBD 2016 Stroke Collaborators 2019), with ischemic stroke comprising ∼85% of all cases (Benjamin et al., 2019). To develop novel diagnostic and therapeutic strategies for ischemic stroke and improve patient outcomes, extensive research has focused on elucidating the mechanisms underlying ischemia–reperfusion (I/R) injury. Emerging evidence suggests that poststroke neuronal damage is not solely attributable to ischemia and hypoxia but is also significantly exacerbated by neuroinflammatory processes.

Erbin, an ERBB2-interacting scaffold protein, is distinguished by its leucine-rich repeat domains. First identified by the Jeanbin team in 2000 (Borg et al., 2000), Erbin is ubiquitously expressed across multiple human and mouse tissues, including the brain, liver, kidneys, spleen, intestine, and skeletal muscle, where it exerts diverse regulatory effects (Huang et al., 2001; Jang et al., 2021). Previous studies have primarily focused on the role of Erbin in tumor progression, extensively investigating its tumor-suppressive and oncogenic functions across various malignancies. However, recent research has increasingly highlighted its involvement in inflammation regulation. During inflammatory responses, Erbin interacts with nucleotide-binding oligomerization domain-containing protein 2 (Nod2), functioning as a novel modulator of the Nod2 signaling pathway (Jang et al., 2021). Additionally, studies indicate that Erbin expression is dynamically regulated by proinflammatory mediators such as lipopolysaccharide (LPS) and tumor necrosis factor-alpha (TNF-α), suggesting its potential role in attenuating inflammatory responses (Shen et al., 2018).

The mitogen-activated protein kinase (MAPK) family primarily consists of three distinct subgroups: extracellular signal-regulated kinase 1/2 (ERK1/2), c-Jun N-terminal kinase (JNK), and p38 MAPK (Kim and Choi, 2015). Previous studies have demonstrated that stress-activated protein kinases, including JNK, p38 MAPK, and ERK, play a deleterious role in cerebral ischemia pathogenesis (Sun and Nan, 2016). Following ischemic insult, phosphorylated p38 MAPK (*p*-p38 MAPK) is prominently expressed in astrocytes, microglia, and neurons within the ischemic brain tissue, underscoring its pivotal role in the inflammatory cascade (Sims and Yew, 2017). The upregulation of inflammatory mediators robustly activates p38 MAPK, leading to blood–brain barrier disruption and the subsequent amplification of proinflammatory cytokine production (Maddahi and Edvinsson, 2010). Notably, inhibition of the p38 MAPK signaling cascade suppresses the release of cytokines such as TNF-α and interleukin-1 beta (IL-1β), thereby exerting neuroprotective effects. Studies on diabetes-induced peripheral neuropathy and epidermal keratinization have further demonstrated that Erbin modulates MAPK pathway activation (Pan and Dobrowsky, 2013). Given the critical role of Erbin in the nervous system and its putative regulatory influence on inflammation, this study employs both in vivo and in vitro models to elucidate the neuroprotective effects of Erbin in ischemic stroke and to explore its potential involvement in MAPK-mediated stroke pathophysiology.

## Materials and Methods

### Animals and ethical considerations

This study utilized male ICR mice (*n* = 165; 8 weeks old; 25–28 g) procured from Charles River Experimental Animal Technology. All animal procedures adhered to the guidelines outlined in the Guide for the Care and Use of Laboratory Animals (NIH publication number 85–23, revised 1996) and were conducted in accordance with ethical standards. The mice were housed under controlled environmental conditions in specific pathogen-free cages at a temperature of 22 ± 2°C, relative humidity of 55 ± 10%, and a 12 h light/dark cycle, with *ad libitum* access to food and water. Social housing was maintained, with five mice per cage. To minimize pain and distress during experimental procedures, mice were killed via cervical dislocation following anesthesia with intraperitoneally administered pentobarbital sodium. The brain tissue was subsequently harvested for analysis. All experimental protocols were approved by the Ethics Committee of the University (Approval No. 2024-LY-053).

### Experimental groups

Mice were randomly allocated into four experimental groups: (1) LV-control–treated sham group (Sham), (2) LV-Erbin overexpression-treated sham group (LV), (3) LV-control–treated I/R group (I/R + NC), and (4) LV-Erbin overexpression-treated I/R group (I/R + LV). Randomization was performed using a predefined randomization table to ensure unbiased allocation. During the experiments, researchers recorded only the identification number of each mouse without noting its group assignment to maintain blinding.

### Middle cerebral artery occlusion (MCAO) model

The MCAO procedure was conducted as previously described. Briefly, a midline cervical incision was made to expose the right common carotid artery, external carotid artery (ECA), and internal carotid artery (ICA). A silicone-coated nylon filament (∼11 mm in length) was inserted into the ICA via the ECA to occlude the right middle cerebral artery. A reduction in cerebral blood flow exceeding 70%, confirmed via Doppler flowmetry, was considered indicative of successful occlusion.

After 50 min of ischemia, the filament was carefully withdrawn to initiate reperfusion. Throughout the procedure, body temperature was maintained at ∼37°C using a heating pad until the mice regained consciousness from anesthesia. In compliance with ethical guidelines and animal welfare regulations, the surgical success rate was ∼85%. Efforts were made to minimize animal use and distress. Mice exhibiting a weight loss exceeding 20% or presenting with severe weakness were excluded from the study.

### Lentiviral vectors construction

The lentiviral (LV) vectors, constructed by OBiO Biotechnology, were designed to overexpress mouse Erbin (transcript variant 3, NM_001289473.1) under the control of the cytomegalovirus promoter and incorporated a green fluorescent protein tag within the vector sequence. The final LV vector titers achieved were 2.3 × 10^8^ TU/ml (Luo et al., 2025; Wang et al., 2025).

### LV vector administration

LV administration was performed 2 weeks prior to MCAO surgery according to the manufacturer's instructions. Following anesthesia, as described previously, a small puncture hole was carefully drilled in the right parietal bone. The LV vector was stereotaxically injected into the right lateral ventricle at the following coordinates relative to the anterior fontanelle: posterior, −0.5 mm; lateral, +1 mm; and ventral, −2.5 mm. All injections were performed using a computer-controlled microinjection pump system. A total volume of 5 µl of LV-control or LV-Erbin-overexpressing vector was delivered at a constant rate of 0.5 µl/min. Following the infusion, the injection needle was left in place for an additional 8 min before being slowly withdrawn.

### Cell lines and cell culture

The HT22 murine hippocampal neuronal cell line was obtained from Beijiasen Biotechnology and cultured in Dulbecco's Modified Eagle Medium (high glucose) supplemented with 10% fetal bovine serum (Invitrogen) and 1% penicillin/streptomycin (Invitrogen). Cells were maintained at 37°C in a humidified incubator with 5% CO_2_.

To establish stable Erbin overexpression, HT22 cells were infected with the LV-Erbin-overexpressing vector or its negative control (LV-control), both obtained from OBiO Biotechnology. Polybrene was used to enhance viral binding to the cell membrane. After 24 h of infection, the culture medium was replaced, and the cells were further incubated for 48 h. According to the instructions, puromycin-containing medium was refreshed every 48 h. Stable infection was verified by immunofluorescence staining and Western blotting after 14 d.

### OGD model and experimental groups

To establish an in vitro oxygen–glucose deprivation (OGD) model, the culture medium of HT22 cells was replaced with a serum-free and glucose-free medium (Invitrogen BRL). The cells were then placed in a specialized hypoxia chamber (Billups-Rothenberg), which was filled with 95% N_2_ and 5% CO_2_. After 4 h of hypoxia, the chamber was opened, and the cells were returned to normal culture conditions.

The cells were divided into four experimental groups: (1) LV-control–treated group (control), (2) LV-Erbin overexpression-treated group (LV), (3) LV-control–treated OGD group (OGD + NC), and (4) LV-Erbin overexpression-treated OGD group (OGD + LV).

### Neurological functional assessment (Longa score)

Neurological function was evaluated 24 h after I/R by a trained laboratory technician using the Longa scoring method (Longa et al., 1989; Deng et al., 2022; Zhang et al., 2024a), as described in previous studies. This method assesses neurological dysfunction based on a predefined scoring scale (scoring standard: 0, normal limb movements, no neurological deficits; 1, when the tail is lifted vertically, the left forepaw is flexed and cannot be straightened; 2, the rat leans to the left when walking flat can be rotated to the left; 3, unstable walking, the entire body dumped to the left; 4, conscious disturbance or unable to walk autonomously).

### Infarct volume measurement

To assess infarct volume, 2,3,5-triphenyltetrazolium chloride (TTC) staining was performed 24 h after MCAO. Briefly, the brain was sectioned into six coronal slices and incubated in 0.2% TTC solution (Sigma-Aldrich) for 30 min at 37°C. The sections were then fixed in 4% paraformaldehyde for 24 h, followed by imaging and analysis using AlphaEase Image Analysis Software (V3.1.2).

### Immunohistochemistry

Mice were anesthetized and perfused transcardially with 4% paraformaldehyde. After dehydration, the brain tissue was embedded in OCT glue and sectioned into 20 µm slices. The sections were washed with PBS and incubated in a blocking solution (Beyotime) for 2 h. For immunostaining, the sections were incubated with primary antibodies against Erbin (1:100, NBP2-56104, Novus Biologicals), Iba1 (1:800, ab283319, Abcam), GFAP (1:800, C9205, Sigma-Aldrich), and NeuN (1:800, NBP 1-92693, Novus Biologicals).

Following incubation with the primary antibody, the tissue sections were incubated with the secondary antibody and processed using a background reduction and autofluorescence quenching kit (Servicebio, G1221). Nuclear counterstaining was performed using an antifade mounting medium containing DAPI (Vector Laboratories). Immunofluorescence images were captured using a confocal microscope (Carl Zeiss).

### Immunoblot analysis

The brain tissue from the right hemisphere (including the ischemic core area and ischemic penumbra area; Kunze et al., 2024; Si et al., 2024) was rapidly excised and stored in liquid nitrogen for subsequent analysis. Protein extraction was performed, and the total protein concentration was determined using a commercial bicinchoninic acid assay kit (Yeasen Biotechnology), followed by subjecting the samples to 8% sodium dodecyl sulfate-polyacrylamide gel electrophoresis (SDS-PAGE) or 4–20% gradient gel (ACE Biotechnology) followed by electro-transfer onto a polyvinylidene fluoride membrane.

The membranes were incubated overnight at 4°C with the primary antibodies against the following proteins: Erbin (1:1,000, NBP2-56104, Novus Biologicals), ERK1/2 (1:500, ET1601–29, Huabio), *p*-ERK1/2 (1:1,000, ET1610–13, Huabio), JNK1/2/3 (1:500, ET1601–28, Huabio), *p*-JNK1/2/3 (1:500, ET1609–42, Huabio), p38 (1:1,000, ET1702–65, Huabio), *p*-p38 (1:1,000, ER2001–52, Huabio), and β-tubulin (1:10,000, ET1602–4, Huabio). To minimize nonspecific binding, we systematically optimized antibody concentrations and blocking conditions.

Detection of protein bands was performed using an enhanced chemiluminescence (ECL) system (Thermo Fisher Scientific). Band density analysis was conducted using AlphaEase Image Analysis Software (V3.1.2).

### Quantitative real-time polymerase chain reaction (qPCR)

Total RNA was extracted using the RNAiso Plus kit (TaKaRa Bio) following the manufacturer's instructions. RNA concentrations were quantified using a NanoDrop 2000 spectrophotometer (Thermo Fisher Scientific).

Reverse transcription was performed using ABScript III RT Master Mix for qPCR (ABclonal Biotechnology) to synthesize complementary DNA (cDNA). qPCR was conducted using 2× Universal SYBR Green (ABclonal Biotechnology) on a 7500 Real-Time PCR system (Applied Biosystems). β-Actin was used as an internal control. The primer sequences were as follows: Erbin, forward, 5′-GCTGTCAAGGTCCTTTAATTCCA-3′; reverse, 5′-TCTTCCGTCTGCAACACTCAG-3′; β-actin, forward, 5′-TGCTATGTTGCCCTAGACTTCG-3′; reverse, 5′-GTTGGCATAGAGGTCTTTACGG-3′. The amplification efficiency was 92.7% for β-actin and 91.8% for Erbin, with correlation coefficients (*R*²) of the standard curves exceeding 0.99 for both genes, which meets standard qPCR requirements.

Primers were synthesized by Sangon Biotech and purified using the PAGE method. All samples were analyzed in duplicate, and relative mRNA expression levels were calculated using the 2^−ΔΔCt^ method to determine fold changes in gene expression.

### Behavioral tests

All behavioral tests, except for the grip strength test, were conducted during daylight hours. Prior to testing, mice were familiarized with the experimental equipment to minimize stress and variability.

On Days 3, 7, and 14 post-I/R, trained laboratory technicians evaluated motor and neurological function using the cylinder test (Vahid-Ansari et al., 2016), pole test (Dunkelmann et al., 2018; Wintz et al., 2023), grip strength test (Reitmeir et al., 2011), and catwalk gait detection (Liu et al., 2021).

Cylinder Test: Mice were placed in a transparent cylinder for 5 min, allowing for unrestricted movement. The percentage of unsuccessful contralateral forelimb placements and the degree of asymmetry were recorded.

Pole Test: Mice were positioned at the top of a 55 cm wooden pole, and video recordings were used to assess turn-around time (T-turn) and descent time. Motor scores were evaluated based on previously described criteria (Dunkelmann et al., 2018; Wintz et al., 2023).

Grip Strength Test: Mice were placed on a grip strength tester, and their tails were gently pulled backward to encourage forelimb grasping of the net frame. The maximum force exerted before slipping was recorded. Multiple trials were conducted to ensure accuracy.

Gait Analysis: The CatWalk XT 10.0 system (Noldus Information Technology) was used for quantitative assessment of gait patterns. Footprint data were analyzed using specialized software to measure parameters, such as footprint length and maximum paw contact area (Liu et al., 2021).

### Enzyme-linked immunosorbent assay (ELISA) for inflammatory factors

Three days postreperfusion, the brain tissue was collected from the injury site. The tissue was immediately immersed in phosphate-buffered saline (PBS) supplemented with protease inhibitors and subsequently homogenized to generate a tissue homogenate using ice-cold PBS at a 1:5 (weight/volume) ratio. The homogenate was centrifuged, and the supernatant was carefully collected. The levels of interleukin (IL)-10 (EM0005, Huabio), IL-1β (EM0001, Huabio), IL-6 (EM0004, Huabio), and TNF-α (EM0010, Huabio) in the supernatant were measured using high-sensitivity commercial ELISA kits according to the manufacturer's instructions.

### Cell counting kit-8 (CCK-8)

Cell viability was measured with a CCK-8 kit (Sangon Biotech). HT22 cells were plated in 96-well plates with three replicates per group. After treatment under OGD conditions, 10 µl of CCK-8 solution was added to each well and incubated at 37°C for 1 h protected from light. The optical density was measured at 450 nm using a microplate reader.

### Statistical analyses

All data are expressed as mean ± SEM, and statistical analyses were performed using GraphPad Prism 9 (GraphPad Software). All data passed the Shapiro–Wilk normality test and were confirmed to conform to the normal distribution (*p* > 0.05). Differences between two groups were analyzed using Student's *t* test, while differences among multiple groups were assessed using one-way ANOVA, followed by Dunnett's multiple-comparison test. Additionally, two-way ANOVA was used to evaluate differences at multiple time points, followed by Tukey's multiple-comparison test, which considered factors such as surgery/OGD and LV vector infection. A *p* value <0.05 was considered statistically significant.

## Results

### Distribution of Erbin in the mouse brain

**Figure 1.**
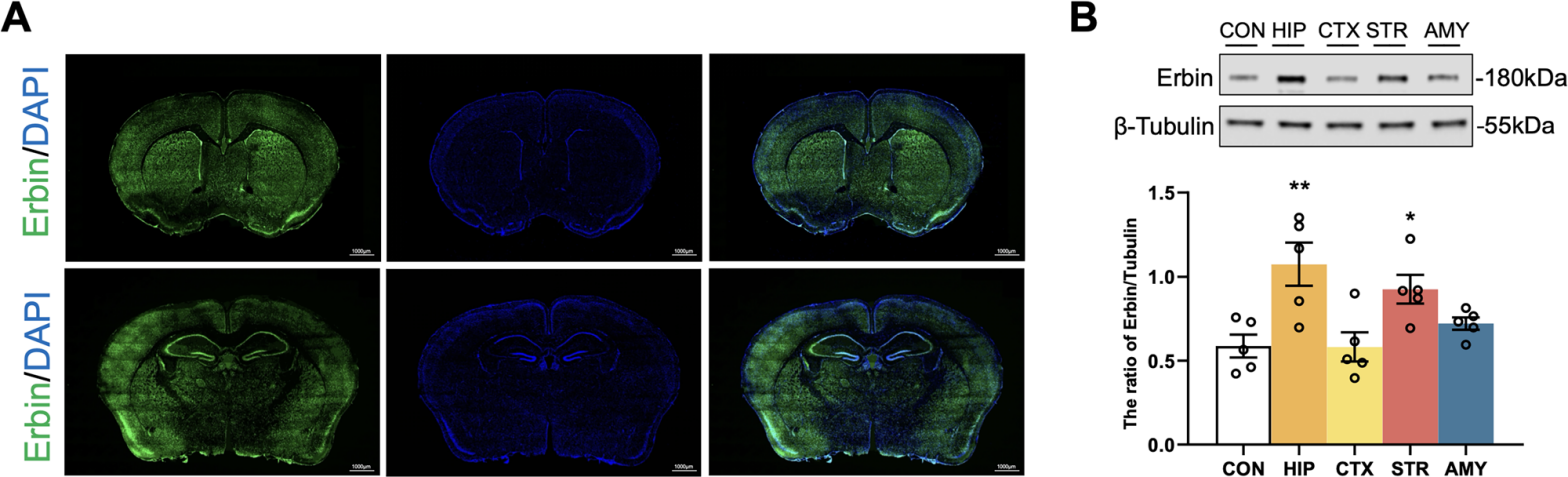
Basal distribution of Erbin in the adult mouse brain. ***A***, Representative fluorescence images of Erbin (green) on coronal sections at the hippocampal and striatal levels. Nuclei are stained with DAPI (blue). Scale bar, 1 mm. ***B***, Quantification of Erbin levels in specific regions. The control group represents the mean intensity from the brain (excluding the cerebellum and brainstem; *n* = 6). Error bars represent means ± SEM. *P < 0.05 and **p < 0.01 versus the control group.

To clarify the expression and distribution characteristics of Erbin in the mouse brain, we performed whole-slide immunofluorescence staining on brain slices under physiological conditions without any treatment. The results showed that Erbin was widely expressed in the brain (Fig. 1*A*). Further regional analysis revealed that the expression abundance of Erbin was higher in the hippocampus and striatum than in other brain regions, and it was also observed in multiple brain regions, indicating that Erbin has extensive expression in the whole brain (Fig. 1*B*; Table 1).

**Table 1.** Statistics associated with Figure 1

Category	Measure	Statistical test	CON	HIP	CTX	STR	AMY	*F* test	*F* test *p* value	CON vs HIP	CON vs CTX	CON vs STR	CON vs AMY
Western blotting	The ratio of Erbin/Tubulin	One-way ANOVA	0.588 ± 0.0682	1.07 ± 0.128	0.582 ± 0.0868	0.926 ± 0.0854	0.721 ± 0.0367	*F*_(3,20)_ = 6.316	*p* = 0.0019	0.0026	ns	0.0388	ns

### Distribution of Erbin in the mouse brains after I/R

**Figure 2.**
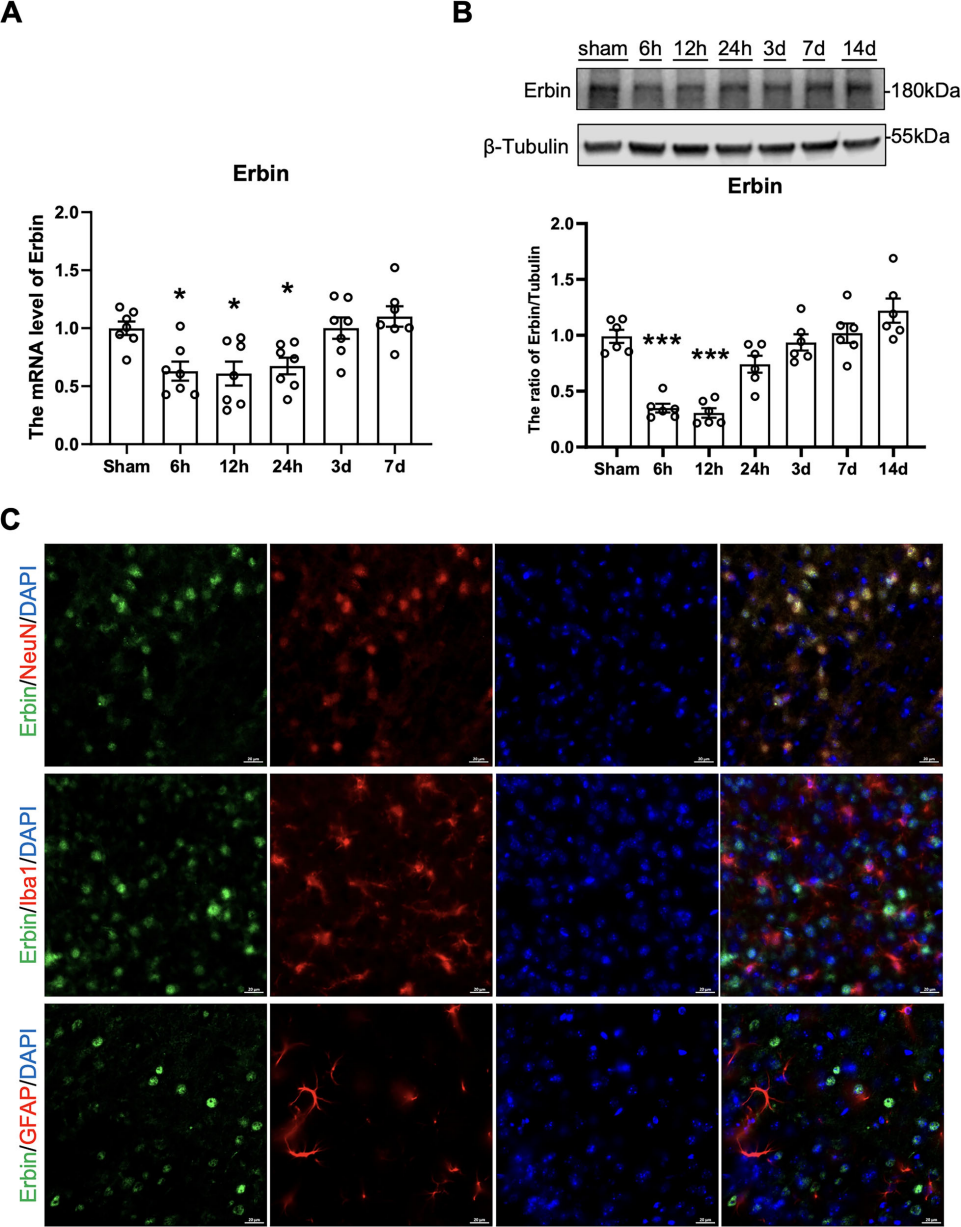
Changes in Erbin and its cellular localization after MCAO. The level of Erbin was decreased after cerebral ischemia/reperfusion injury. ***A***, The Q-PCR shows the mRNA level of Erbin after MCAO (*n* = 7). ***B***, Representative Western blot staining of Erbin and statistical analysis of the relative intensity (*n* = 6). ***C***, Cellular localization of Erbin (green) in the cerebral cortex within the ischemic penumbra at 24 h after reperfusion. The image was taken from I/R model mice without virus injection. Brain sections were stained with Erbin (green) and cell markers (NeuN, Iba1, GFAP, red) to show colocalization of endogenous Erbin with neurons, microglia, and astrocytes. Scale bar represents 20 µm. Error bars represent means ± SEM. *P < 0.05 and ***p < 0.001 versus the sham group.

To gain deeper insight into the role of Erbin in I/R injury, brain tissues were collected at multiple time points to quantify Erbin expression. Both qPCR (Fig. 2*A*; Table 2) and Western blot analysis (Fig. 2*B*; Table 2) produced concordant results, demonstrating a progressive decline in Erbin levels following I/R, with the lowest expression observed between 6 and 12 h. Subsequently, Erbin expression gradually returned to baseline levels within 3–14 d. Additionally, immunofluorescence staining was performed to elucidate the cellular localization of Erbin within the affected brain regions. Colocalization analysis using specific cellular markers identified Erbin expression predominantly in neurons, with negligible expression detected in microglia and astrocytes following I/R (Fig. 2*C*).

**Table 2.** Statistics associated with Figure 2

Category	Measure	Statistical test	Sham	6 h	12 h	24 h	3 d	7 d	14 d	*F* test	*F* test *p* value	Sham vs 6 h	Sham vs 12 h	Sham vs 24 h	Sham vs 3 d	Sham vs 7 d	Sham vs 14 d
qPCR	The mRNA level of Erbin	One-way ANOVA	0.9994 ± 0.05854	0.6296 ± 0.08254	0.6096 ± 0.1036	0.6745 ± 0.07213	1.001 ± 0.09109	1.101 ± 0.08852	–	*F*_(5,36)_ = 6.921	*p* = 0.0001	0.0155	0.0100	0.0392	ns	ns	–
Western blotting	The ratio of Erbin/Tubulin	One-way ANOVA	0.9906 ± 0.05882	0.3487 ± 0.03900	0.3056 ± 0.04248	0.7415 ± 0.07558	0.9366 ± 0.07232	1.019 ± 0.08663	1.221 ± 0.1086	*F*_(6,35)_ = 23.06	*p* < 0.0001	<0.0001	<0.0001	ns	ns	ns	ns

### Overexpression of Erbin mitigates neurological deficits following I/R injury

**Figure 3.**
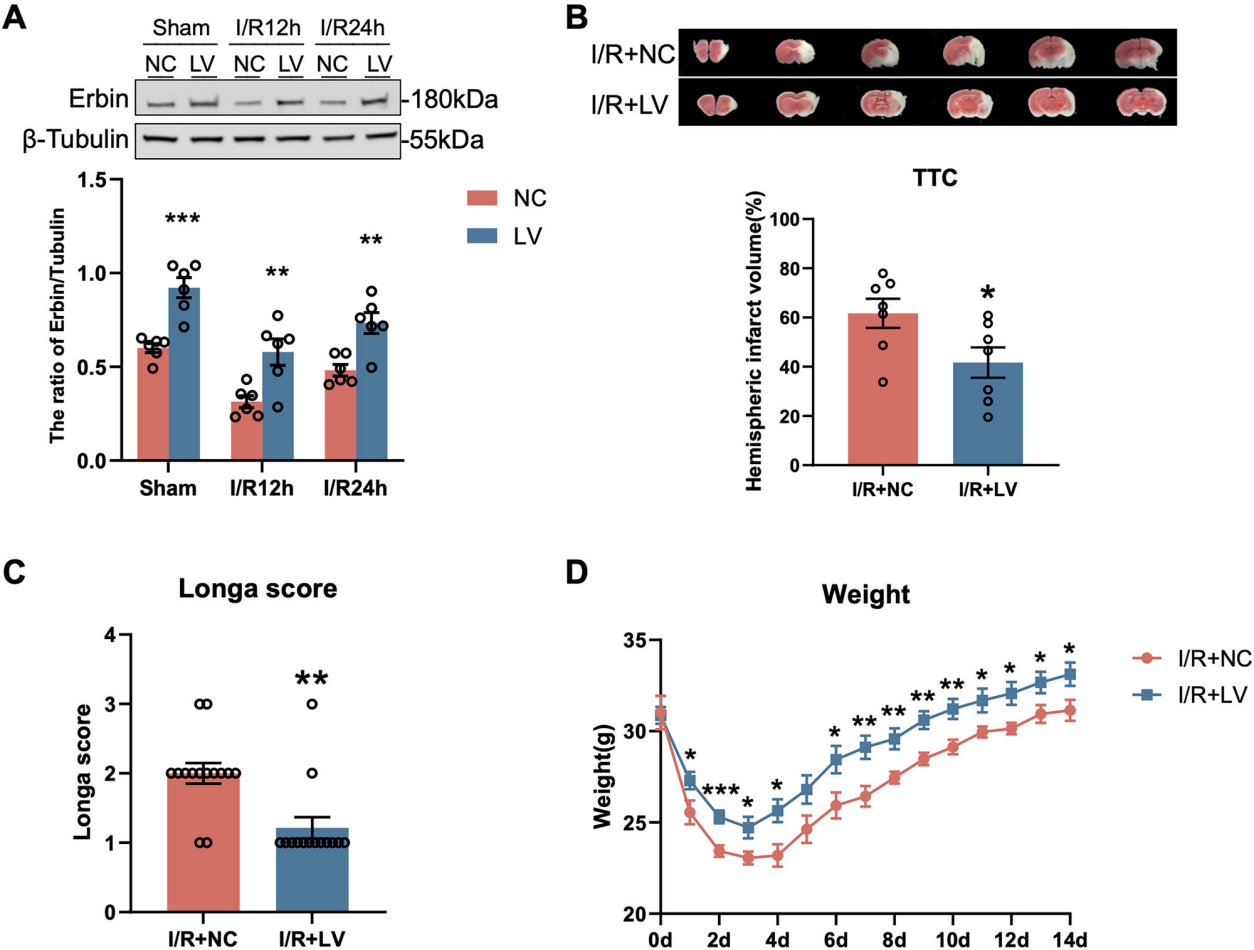
LV-Erbin infection reduced infarct volume and decreased neurological deficits after MCAO. ***A***, Representative Western blot images and quantification of Erbin protein levels before and after I/R (*n* = 6). Error bars represent means ± SEM. **P < 0.01 and ***p < 0.001 versus NC group (related data can be found in Extended Data Fig. 3-1). ***B***, Representative imagines of TTC-stained brain sections were shown and quantitative analysis of cerebral infarct volume after LV infection was performed (*n* = 7). ***C***, The protective effects of LV-Erbin on the Longa score at 24 h after reperfusion were evaluated (*n* = 14). ***D***, The weight change of mice after MCAO (*n* = 10). Bars represent means ± SEM. *P < 0.05 and **p < 0.01 versus I/R + NC group.

To investigate the role of Erbin in I/R injury, an LV vector was constructed to achieve stable overexpression of Erbin. The Erbin-expressing LV vector was injected into the right lateral ventricle, while control mice received an injection of an empty LV vector (related data can be found in Extended Data Fig. 3-1). Subsequently, mice underwent MCAO followed by reperfusion. Western blot showed that lentivirus-mediated Erbin overexpression established high baseline levels before modeling. After I/R, Erbin levels in the overexpression group declined at 12 and 24 h but remained markedly higher than in controls (Fig. 3*A*; Table 3). This indicates that the LV strategy maintains high Erbin expression across the pre- and postinjury periods. TTC staining, performed 24 h post-MCAO, revealed a significant reduction in infarct volume in mice treated with the Erbin LV vector compared with the LV-control group. Quantitative analysis confirmed a statistically significant decrease in brain injury volume following Erbin overexpression (Fig. 3*B*; Table 3).

10.1523/ENEURO.0089-25.2026.f3-1 Figure 3-1 **A.** Lentivirus-mediated Erbin overexpression in the mouse brain. Western blot analysis (top) and corresponding densitometric quantification (bottom) of ipsilateral brain collected two weeks after intracerebroventricular injection. Mice were treated with either an Erbin-expressing lentivirus (LV), a control empty lentivirus (NC), or were left as Sham. The control empty lentivirus did not alter endogenous Erbin levels. Data were normalized to β-Tubulin as a loading control and are presented as mean ± SEM (n=4 per group). ***P < 0.001 versus the LV group. **B.** In vivo distribution of the Erbin-overexpressing lentivirus. Immunofluorescence image of a coronal brain section prepared two weeks after intracranial injection of the GFP-tagged lentivirus into the right lateral ventricle. The image shows robust viral transduction, as indicated by GFP signal (green), with prominent expression observed in the ipsilateral cortex, striatum, and hippocampus. This result serves as a visual demonstration of viral expression and distribution under basal conditions prior to injury. Download Figure 3-1, TIF file.

**Table 3.** Statistics associated with Figure 3

Category	Measure	Statistical test	Group	NC	LV	NC vs LV
Western blotting	The ratio of Erbin/tubulin	*T* test	Sham	0.6001 ± 0.02413	0.9216 ± 0.05355	<0.001
			I/R12h	0.3141 ± 0.03196	0.5789 ± 0.07030	0.006
			I/R24h	0.4821 ± 0.03091	0.7342 ± 0.05553	0.003

Category	Measure	Statistical test	I/R + NC	I/R + LV	I/R + LV vs I/R + NC
TTC	Hemispheric infarct volume(%)	*T* test	61.7 ± 5.91	41.7 ± 6.16	0.04
Infarct volume measurement	Longa score	*T* test	2.00 ± 0.148	1.21 ± 0.155	0.001

Neurological function was assessed 24 h post-MCAO using a standardized neurological scoring system, where higher scores indicate greater impairment. Mice receiving the Erbin LV vector exhibited significantly higher neurological scores than those in the LV-control group (Fig. 3*C*; Table 3). Additionally, body weight changes were monitored after MCAO. Both groups experienced a progressive decline in body weight, reaching the lowest point within 3 d post-MCAO, followed by gradual recovery over 14 d. Notably, mice treated with the Erbin LV vector exhibited a lesser degree of weight loss and maintained a consistently higher body weight compared with the LV-control group throughout the 14 d observation period (Fig. 3*D*). These findings indicate that Erbin overexpression exerts a neuroprotective effect against cerebral I/R injury.

### Erbin enhances neurological recovery during the postreperfusion period

**Figure 4.**
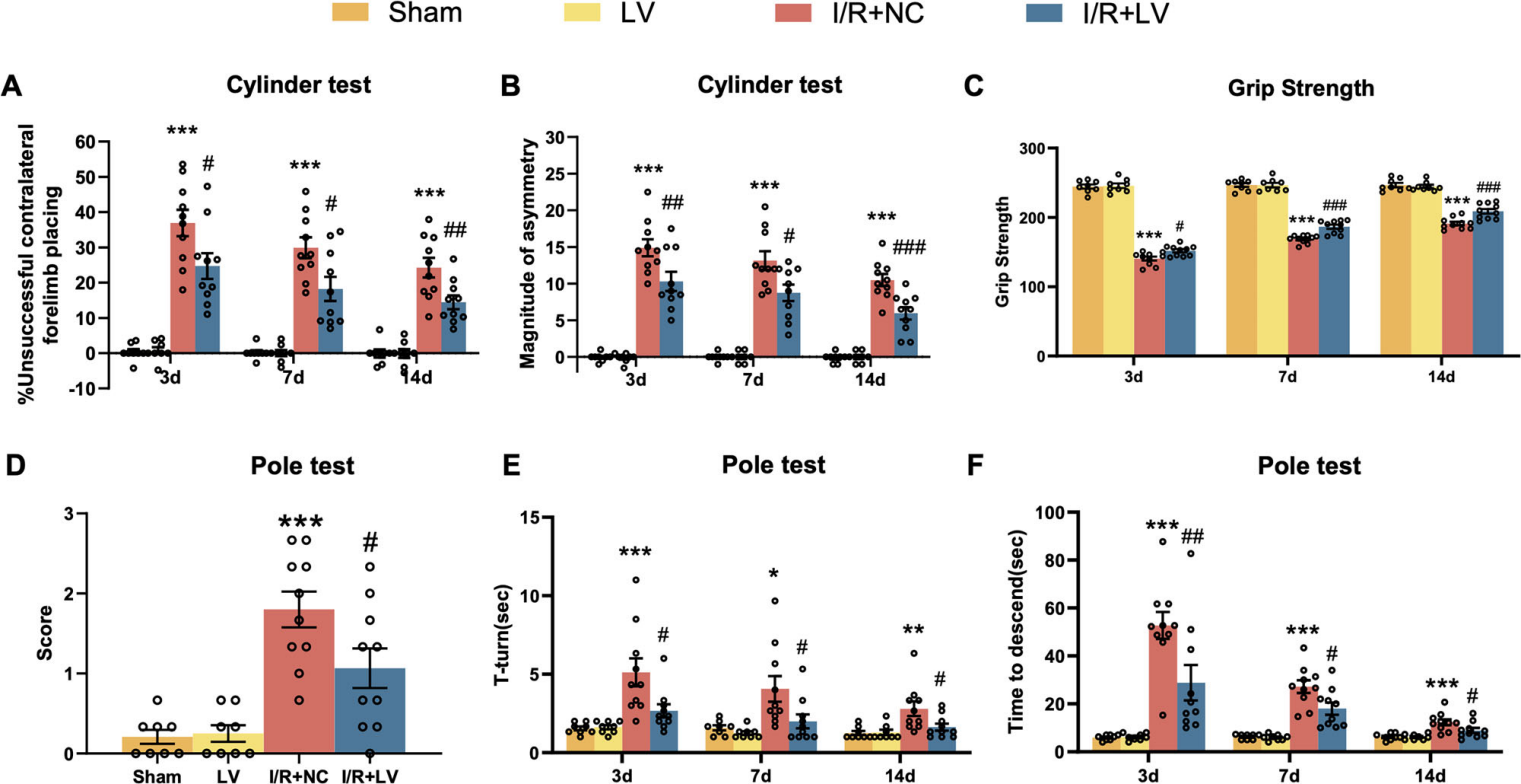
LV-Erbin infection improves behavioral performance after MCAO. Cylinder test (***A*, *B***; *n* = 10), grip strength test (***C***; *n* = 8), and pole test (***D–F***; *n* = 10) analysis at different time points (related data can be found in Extended Data Fig. 4-1). Error bars represent means ± SEM. *P < 0.05, **p < 0.01, and ***p < 0.001 versus sham group, *^#^p* < 0.05, *^##^p* *<* 0.01, and *^###^p* < 0.001 versus I/R + NC group.

To comprehensively assess neural function following reperfusion, the mild cylinder test was employed to evaluate asymmetric forelimb use in mice. According to the protocol, the number of exploratory movements using the left and right forelimbs was recorded for statistical analysis. As shown in Figure 4, *A* and *B* (Extended Data Fig. 4-1), in mice without I/R injury, LV administration did not significantly affect forelimb symmetry, with the difference and proportion of asymmetric use between the left and right forelimbs remaining close to zero. However, in mice subjected to cerebral I/R injury, those treated with the Erbin LV vector exhibited a significantly lower difference and ratio in left and right forelimb use on Days 3, 7, and 14 postreperfusion compared with the LV-control group.

10.1523/ENEURO.0089-25.2026.f4-1 Figure 4-1 Statistics associated with Figure 4. Data are presented as mean ± SEM for four experimental groups: Sham, LV, I/R+NC, and I/R+LV. Behavioral assessments included the cylinder test (forelimb placing and asymmetry), grip strength, and the pole test (score, T-turn time, and descent time). Two-way ANOVA was applied as indicated. Statistical comparisons between groups are shown in the table, with significance denoted by *P* values or “ns” (not significant). Download Figure 4-1, DOC file.

Consistent with these findings, results from the grip strength test (Fig. 4*C*; Extended Data Fig. 4-1) demonstrated that mice in the MCAO and reperfusion groups treated with the Erbin LV vector exhibited superior strength preservation and recovery compared with the LV-control group. In contrast, LV administration had no significant effect on muscle strength in the sham surgery group.

To further evaluate fine motor function and balance, the pole test was conducted. According to the protocol, a higher score indicated greater difficulty in descending, reflecting more severe neurological impairment. Additionally, the time required for mice to turn at the top of the pole (T-turn) and the total descent time were recorded. Results showed that mice with I/R injury exhibited significantly higher scores than those in the sham surgery group, while those in the LV-Erbin group performed better than the LV-control group (Fig. 4*D*; Extended Data Fig. 4-1). Furthermore, at multiple time points post-MCAO and reperfusion, the LV-Erbin group demonstrated significantly shorter T-turn times (Fig. 4*E*; Extended Data Fig. 4-1) and faster descent times (Fig. 4*F*; Extended Data Fig. 4-1) compared with the LV-control group.

These findings suggest that Erbin overexpression promotes functional recovery and mitigates neurological damage following cerebral I/R injury.

**Figure 5.**
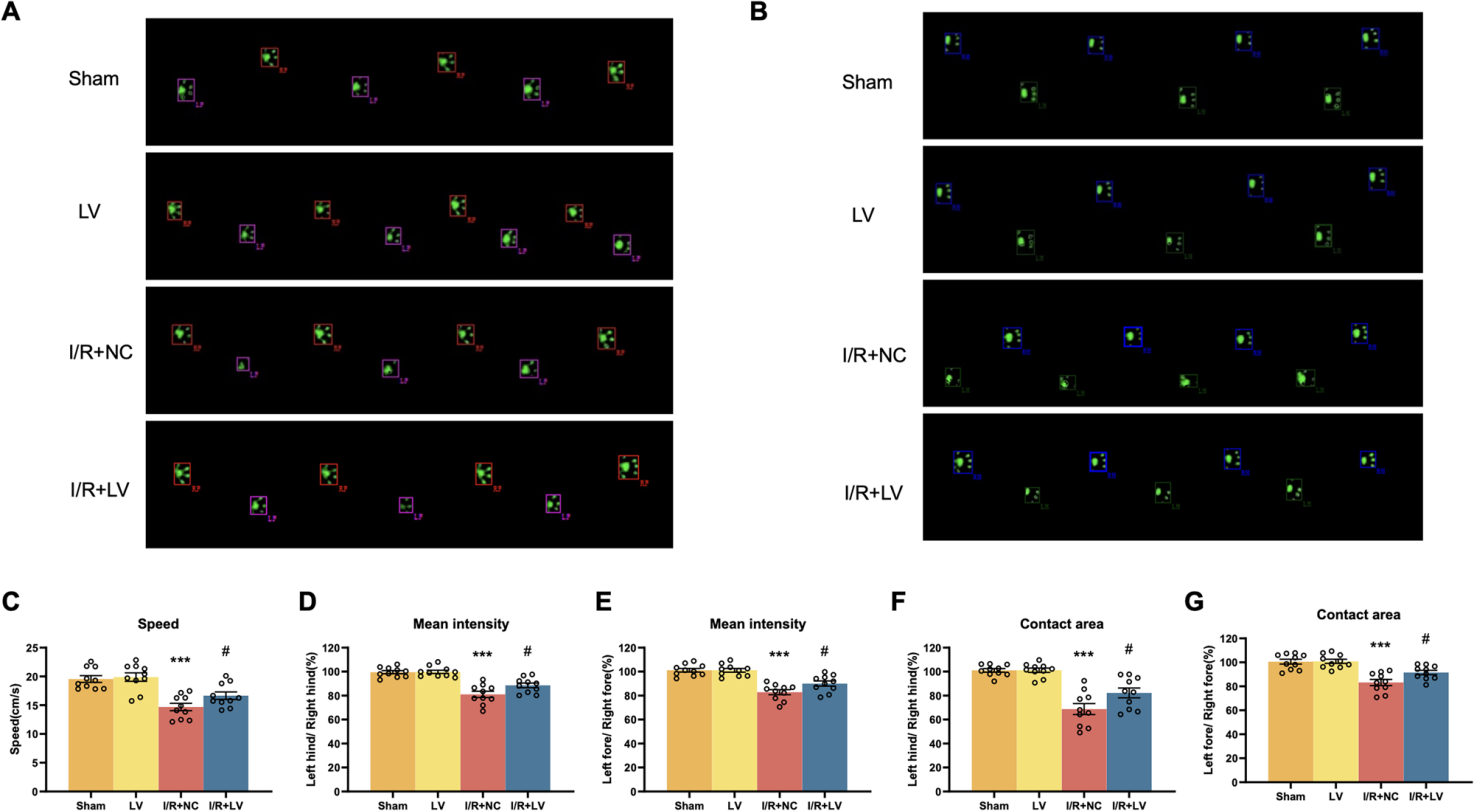
LV Erbin infection improves gait dysfunction in mice after MCAO. The typical footprint of a mouse gait is shown (***A*, *B***), with the right forefoot (RF) marked in red, the left forefoot (LF) marked in purple, the right hind foot (RH) marked in blue, and the left hind foot (LH) marked in green. Speed (***C***), mean intensity (***D–E***), and the contact area (***F–G***) are presented between I/R + NC and I/R + LV groups (*n* = 10). Error bars represent means ± SEM. ***P < 0.001 versus the sham group; *^#^p* < 0.05 versus I/R + NC group.

Gait analysis was conducted to assess behavioral changes associated with I/R injury and to comprehensively evaluate locomotor function in mice. Parameters, including walking speed, mean intensity, and contact area, were analyzed. In the gait analysis system, the I/R + LV group exhibited significantly faster walking speed, higher mean intensity in the left limb, and a larger contact area compared with the I/R + NC group. No significant differences were observed between the sham group and the LV group. These findings suggest that Erbin upregulation enhances neurological recovery in I/R mice and provides a protective effect against cerebral I/R injury (Fig. 5, Table 4).

**Table 4.** Statistics associated with Figure 5

Category	Measure	Statistical test	Sham	LV	I/R + NC	I/R + LV	*F* test (LV vector)	*F* test *p* value (LV vector)	Sham vs LV	Sham vs I/R + NC	Sham vs I/R + LV	I/R + NC vs I/R + LV
Behavioral tests	Speed	Two-way ANOVA	19.56 ± 0.5763	19.88 ± 0.7303	14.69 ± 0.6398	17.16 ± 0.5142	*F*_(1,36)_ = 5.087	*p* = 0.0303	ns	<0.0001	0.0462	0.0376
	Mean intensity hind	Two-way ANOVA	99.52 ± 1.290	99.83 ± 1.340	81.05 ± 2.564	88.50 ± 1.981	*F*_(1,36)_ = 4.312	*p* = 0.0450	ns	<0.0001	0.0010	0.0371
	Mean intensity fore	Two-way ANOVA	101.2 ± 1.499	101.1 ± 1.548	82.87 ± 2.122	90.10 ± 2.191	*F*_(1,36)_ = 3.668	*p* = 0.0634	ns	<0.0001	0.0009	0.0454
	Contact area hind	Two-way ANOVA	101.0 ± 1.463	101.2 ± 1.776	68.75 ± 4.574	82.23 ± 4.117	*F*_(1,36)_ = 4.280	*p* = 0.0458	ns	<0.0001	0.0014	0.0307
	Contact area fore	Two-way ANOVA	100.6 ± 2.028	100.9 ± 1.767	83.20 ± 2.528	91.56 ± 1.812	*F*_(1,36)_ = 4.447	*p* = 0.0420	ns	<0.0001	0.0186	0.0327

### Erbin exhibits a neuroprotective role in an OGD-induced neuronal injury model

**Figure 6.**
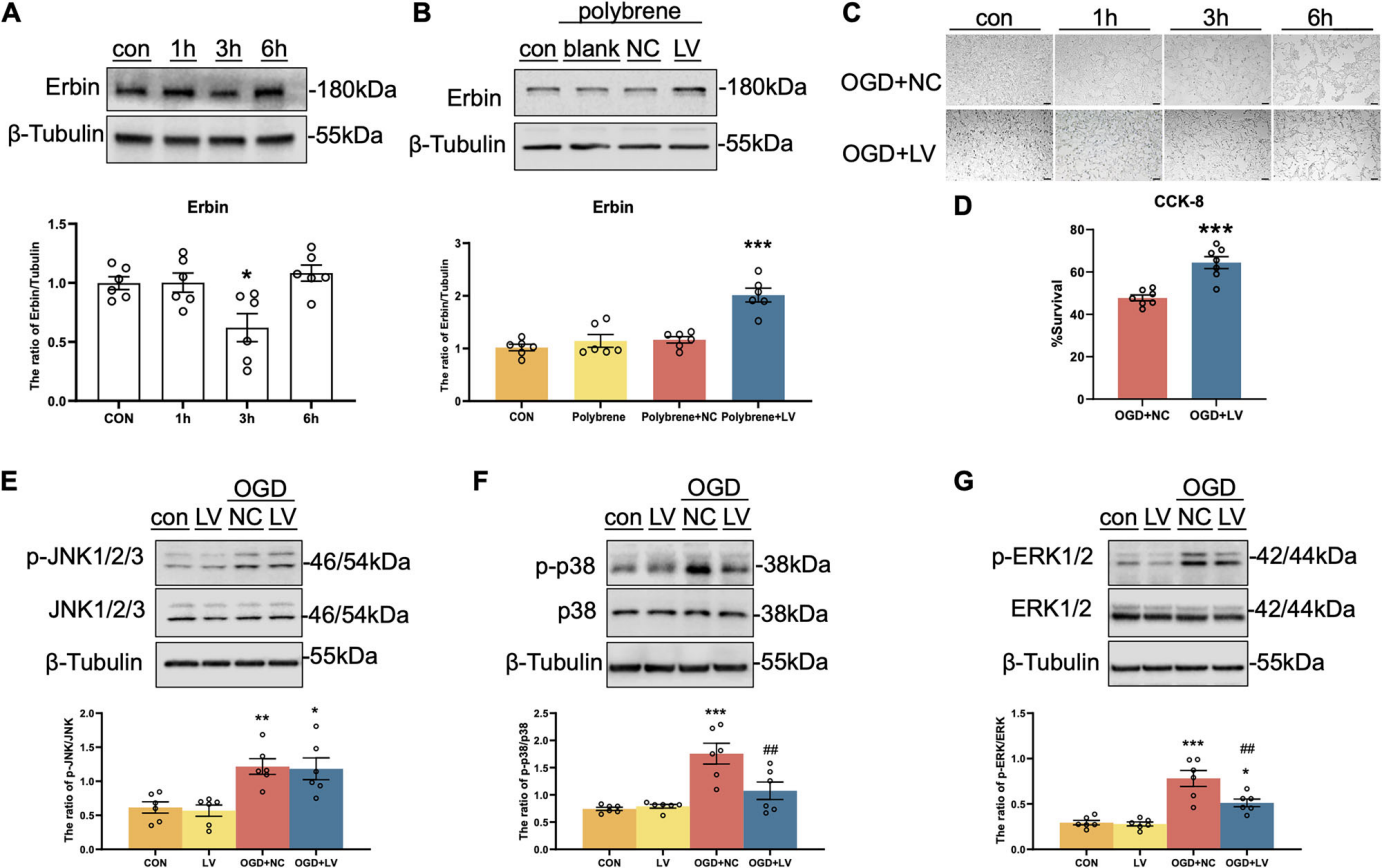
Erbin levels decreased in HT22 after OGD and exerted a protective effect. ***A***, Erbin levels in HT22 cells collected at different time points after OGD. **P < 0.01 versus control group (*n* = 8). ***B***, Erbin levels in the stable Erbin-overexpressing cell lines generated using LV infection (*n* = 6), ***p < 0.001 versus the control group. ***C***, Representative images of cell density taken at different time points before and after OGD; scale bar, 200 µm. ***D***, Survival rate of HT22 cells detected using CCK-8 assay 6 h after OGD (*n* = 6); ***p < 0.001 versus OGD + NC group. Bars represent means ± SEM. Representative Western blot staining of JNK1/2/3 (***E***), p38 (***F***), and ERK1/2 (***G***) and their phosphorylated forms and statistical analysis of the relative ratio (*n* = 6). Error bars represent means ± SEM. *P *<* *0.05*, **p < 0.01, and ***p < 0.001 versus the control group; *^##^p* *<* 0.01 versus the OGD + NC group.

To further elucidate the protective mechanisms of Erbin in cerebral I/R injury, this study utilized an OGD model in HT22 cells, given that prior immunofluorescence analysis indicated Erbin's predominant localization in neurons. To assess the temporal expression pattern of Erbin following OGD, we collected samples at multiple time points (1, 3, and 6 h). Consistent with in vivo findings, Erbin levels initially declined post-OGD before gradually returning to baseline levels (Fig. 6*A*; Table 5).

**Table 5.** Statistics associated with Figure 6*A* and *B*

Category	Measure	Statistical test	CON	1 h	3 h	6 h	*F* test	*F* test *p* value	CON vs 1 h	CON vs 3 h	CON vs 6 h	1 h vs 3 h	3 h vs 6 h
Western blotting	The ratio of Erbin/tubulin	One-way ANOVA	0.998 ± 0.0542	1.00 ± 0.0808	0.620 ± 0.119	1.08 ± 0.0680	*F*_(3,20) _= 6.09	*p* = 0.0041	ns	0.0225	ns	0.0210	0.0046
			CON	Polybrene	Polybrene + NC	Polybrene + LV	*F* test	*F* test *p* value	CON versus Polybrene	CON versus Polybrene + NC	CON versus Polybrene + LV	Polybrene versus Polybrene + NC	Polybrene + NC versus Polybrene + LV
			1.018 ± 0.06209	1.145 ± 0.1204	1.166 ± 0.06105	2.014 ± 0.1306	*F*_(3,20)_ = 21.35	*p* < 0.0001	<0.0001	ns	<0.0001	ns	<0.0001

To establish a stable Erbin-overexpressing neuronal cell line, we transduced HT22 cells with a LV vector carrying Erbin, with polybrene used to enhance transduction efficiency. Western blot analysis confirmed that Erbin expression was significantly elevated in LV-Erbin–infected cells, while polybrene itself had no impact on Erbin expression (Fig. 6*B*; Table 5). This stable cell line was subsequently employed for OGD experiments. The results demonstrated that Erbin-overexpressing cells exhibited higher cell density compared with the LV-control group at all time points post-OGD (Fig. 6*C*). Furthermore, CCK-8 assays performed at 6 h post-OGD revealed a significantly higher survival rate in Erbin-overexpressing cells compared with control cells (Fig. 6*D*; Table 6), indicating that Erbin overexpression enhances neuronal viability and confers protection against OGD-induced damage.

**Table 6.** Statistics associated with Figure 6*D*

Category	Measure	Statistical test	I/R + NC	I/R + LV	I/R + LV vs I/R + NC
CCK-8	%Survival	*T* test	47.8 ± 1.37	64.4 ± 2.81	<0.001

Previous studies have established that Erbin negatively regulates the activation of the MAPK signaling pathway, particularly the ERK pathway (Huang et al., 2003). To determine whether Erbin retains this regulatory role in neurons following cerebral I/R injury, this study assessed the phosphorylation status of key MAPK pathway components, including p38, ERK1/2, and JNK1/2/3. Western blot analysis of surviving cells at 6 h post-OGD revealed that basal levels of phosphorylated ERK (*p*-ERK), JNK, and phosphorylated p38 (*p*-p38) were low under normal conditions. Following OGD, *p*-ERK levels were significantly reduced in Erbin-overexpressing cells, mirroring Erbin's inhibitory effect on ERK observed in other disease models. Notably, *p*-p38 levels were also markedly lower in the LV-Erbin group compared with the LV-control group, whereas *p*-JNK levels remained unchanged. These findings suggest that Erbin mitigates I/R injury by inhibiting the MAPK signaling cascade in neurons, specifically by downregulating p38 and ERK phosphorylation, while exerting no significant effect on JNK activation (Fig. 6*E–G*; Table 7).

**Table 7.** Statistics associated with Figure 6*E*–*G*

Category	Measure	Statistical test	CON	LV	OGD + NC	OGD + LV	*F* test (LV vector)	*F* test *p* value (LV vector)	CON vs LV	CON vs OGD + NC	CON vs OGD + LV	OGD + NC vs OGD + LV
Western blotting	The ratio of *p*-JNK/JNK	Two-way ANOVA	0.6152 ± 0.08342	0.5689 ± 0.08310	1.216 ± 0.1158	1.183 ± 0.1599	*F*_(1,20)_ = 0.1210	*p* = 0.7316	ns	0.0071	0.0114	ns
	The ratio of *p*-p38/p38	Two-way ANOVA	0.7435 ± 0.02933	0.7912 ± 0.03467	1.758 ± 0.1905	1.077 ± 0.1600	*F*_(1,20)_ = 6.279	*p* = 0.0210	ns	<0.0001	ns	0.0056
	The ratio of *p*-ERK/ERK	Two-way ANOVA	0.2946 ± 0.02516	0.2793 ± 0.02249	0.7814 ± 0.08789	0.5124 ± 0.04232	*F*_(1,20)_ = 7.592	*p* = 0.0122	ns	<0.0001	0.0341	0.0073

### Erbin mitigates I/R injury by regulating the MAPK pathway

**Figure 7.**
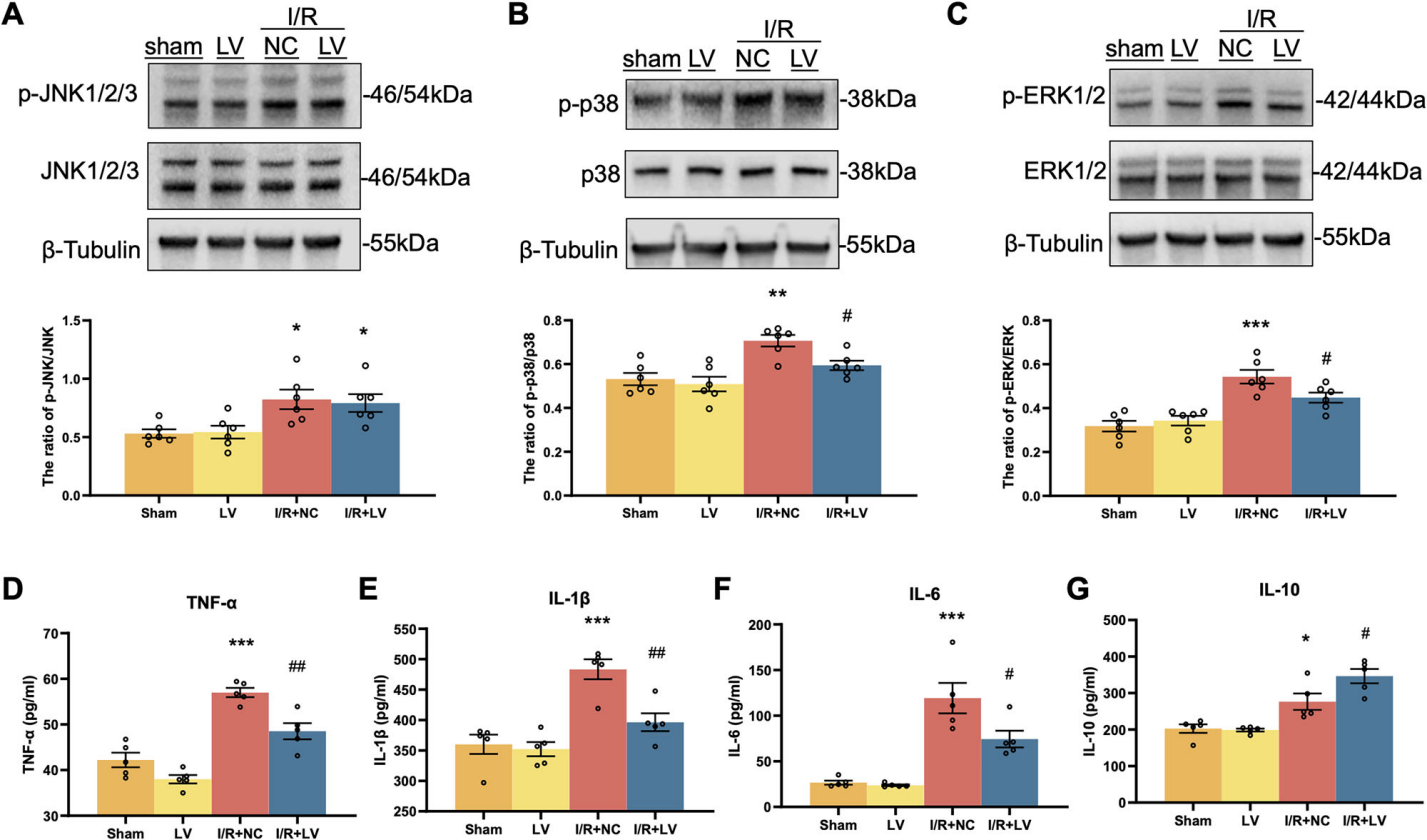
Erbin overexpression mitigates I/R injury via MAPK and anti-inflammatory pathways. Western blot analysis of MAPK signaling pathway activation in mouse brain tissues after I/R injury. The expression levels of phosphorylated (*p*-) and total JNK1/2/3 (***A***), p38 (***B***), and ERK1/2 (***C***) were normalized to tubulin. Data are presented as the ratio of phosphorylated to total protein levels (*n* = 6). ***D–G***, ELISA analysis of inflammatory cytokine levels in mouse brain tissues, including TNF-α, IL-1β, IL-6, and IL-10 (*n* = 5). Error bars represent means ± SEM. *P *<* *0.05*, **p *<* *0.01*, and ***p *<* *0.001* versus the control group. *^#^P* < 0.05; *^##^p* < 0.01 versus the OGD + NC group.

To investigate the role of Erbin in modulating the MAPK signaling pathway following cerebral I/R injury, we assessed protein expression levels in mouse brain tissues on the third day postinjury. Western blot analysis revealed that I/R injury significantly activated the JNK1/2/3, p38, and ERK1/2 signaling pathways, as indicated by increased phosphorylation of these proteins compared with the sham surgery group (Fig. 7*A*–*C*; Table 8). Specifically, the ratios of *p*-JNK1/2/3 to total JNK1/2/3, *p*-p38 to total p38, and *p*-ERK1/2 to total ERK1/2 were significantly elevated in the I/R + NC group relative to the sham group. Notably, in the I/R + LV group, where Erbin was overexpressed, phosphorylation levels of *p*-ERK and p38 were markedly reduced compared with the I/R + NC group, while *p*-JNK levels remained unchanged.

**Table 8.** Statistics associated with Figure 7

Category	Measure	Statistical test	Sham	LV	I/R + NC	I/R + LV	*F* test (LV vector)	*F* test *p* value (LV vector)	Sham vs LV	Sham vs I/R + NC	Sham vs I/R + LV	I/R + NC vs I/R + LV
	The ratio of *p*-JNK/JNK	Two-way ANOVA	0.5306 ± 0.03581	0.5431 ± 0.05497	0.8228 ± 0.08391	0.7920 ± 0.07601	*F*_(1,20)_ = 0.01935	*p* = 0.8908	ns	0.0235	0.0473	ns
	The ratio of *p*-p38/p38	Two-way ANOVA	99.52 ± 1.290	99.83 ± 1.340	81.05 ± 2.564	88.50 ± 1.981	*F*_(1,20)_ = 1.930	*p* = 0.0450	ns	0.0013	ns	0.0435
	The ratio of *p*-ERK/ERK	Two-way ANOVA	0.3182 ± 0.02424	0.3431 ± 0.02183	0.5433 ± 0.03081	0.4482 ± 0.02307	*F*_(1,20)_ = 1.930	*p* = 0.1801	ns	<0.0001	0.0081	0.0368
	TNF-α	Two-way ANOVA	42.22 ± 1.604	38.01 ± 0.9190	56.99 ± 1.018	48.52 ± 1.765	*F*_(1,16)_ = 21.26	*p* = 0.0003	ns	<0.0001	0.0239	0.0025
	IL-1β	Two-way ANOVA	360.2 ± 15.88	352.3 ± 11.57	483.4 ± 16.37	396.5 ± 14.58	*F*_(1 16)_ = 10.39	*p* = 0.0053	ns	0.0001	ns	0.0036
	IL-6	Two-way ANOVA	26.73 ± 2.252	23.82 ± 0.9326	119.2 ± 16.64	74.42 ± 9.130	*F*_(1,16)_ = 6.214	*p* = 0.0240	ns	<0.0001	0.0135	0.0207
	IL-10	Two-way ANOVA	100.6 ± 2.028	100.9 ± 1.767	83.20 ± 2.528	91.56 ± 1.812	*F*_(1,16)_ = 4.180	*p* = 0.0577	ns	0.0255	<0.0001	0.0339

Furthermore, the concentrations of key inflammatory cytokines, including TNF-α, IL-1β, IL-6, and IL-10, were quantified (Fig. 7*D*–*G*; Table 8). The results demonstrated a significant upregulation of these cytokines following I/R injury compared with the sham group. Conversely, in the I/R + LV group, cytokine levels were markedly reduced relative to the I/R + NC group, with significant decreases observed in TNF-α, IL-1β, and IL-6, alongside an elevation in the protective cytokine IL-10. These findings indicate that I/R injury activates the MAPK signaling pathways, driving a robust inflammatory response. Notably, Erbin overexpression mitigates this inflammatory cascade via the ERK and p38 pathways, underscoring its neuroprotective potential.

Collectively, these results provide valuable insights into the neuroprotective role of Erbin in cerebral I/R injury, highlighting its potential as a therapeutic target for mitigating MAPK-mediated neuronal damage and inflammation.

## Discussion

The MAPK signaling pathway plays a central role in the inflammatory response to I/R injury (Jiang et al., 2014), serving as a key regulator of neuroinflammation (Kimelberg et al., 2006). Activation of MAPK leads to an overproduction of proinflammatory cytokines, contributing to neuronal damage (Himaya et al., 2011). Among the MAPK subfamilies, p38 MAPK is particularly associated with cytokine generation, demonstrating a stronger correlation with inflammatory processes than ERK (Kim et al., 2005; Xu et al., 2021). The present study provides compelling evidence that Erbin upregulation inhibits the MAPK signaling pathway, thereby reducing inflammation and oxidative stress and mitigating neurological deficits in a mouse model of ischemic stroke. Our findings indicate that Erbin expression is downregulated in the brain tissue following MCAO-induced damage and in neurons subjected to OGD. Importantly, Erbin overexpression improves neurological outcomes post-I/R injury, enhances stroke recovery, and promotes neuronal survival.

Based on our experimental findings, the temporal dynamics of this protective effect can be outlined as follows: during the early phase postreperfusion (Days 0–1), neuroprotection is primarily mediated by the overexpression of Erbin via lentivirus. As we move into the middle and late stages (Days 3–14), we propose that the gradual recovery of both exogenous overexpression and endogenous Erbin works in tandem to facilitate the ongoing repair and remodeling of neural function. Consequently, lentivirus-mediated overexpression serves as the cornerstone for initiating and sustaining the entire protective process, while the restoration of endogenous Erbin may offer crucial synergistic support in the later stages. Mechanistically, Erbin mediates its neuroprotective effects by attenuating MAPK pathway activation, primarily through the suppression of ERK and p38 phosphorylation. This key finding underscores Erbin's potential as a therapeutic target for mitigating neuroinflammation and neuronal injury in ischemic stroke.

Initially characterized for its interaction with ERBB2, Erbin contains distinct leucine-rich repeat and PDZ domains that facilitate its interaction with a diverse array of intracellular proteins. These structural attributes suggest that Erbin may play a broader regulatory role in intracellular signaling networks, extending beyond its modulation of the MAPK pathway (Jang et al., 2021). Erbin plays a critical role in the surface expression of AMPA receptors in cortical parvalbumin-positive interneurons, contributing to dendritic growth and branching in hippocampal neurons (Arikkath et al., 2008).

Beyond its neuroregulatory functions, Erbin also plays a key role in modulating neuroinflammation. Previous studies have demonstrated that neuronal expression of various factors can modulate inflammatory responses through the regulation of specific signaling pathways, suggesting a critical role of neuron-derived molecules in poststroke neuroinflammation (Li et al., 2016; Puig et al., 2018). In this study, we further demonstrated that Erbin, which is predominantly expressed in neurons, significantly alleviates the inflammatory response after cerebral ischemia when overexpressed. Following ischemic injury, the expression of multiple cytokines shifts from baseline to elevated levels as part of the endogenous repair mechanism (Baba Ahmadi et al., 2024; Shi et al., 2024; Tang et al., 2025). Notably, Erbin overexpression enhanced this adaptive process, underscoring its role in amplifying anti-inflammatory signaling and facilitating neural repair. Erbin is regulated by proinflammatory mediators such as muramyl dipeptide, LPS, and TNF-α, and it interacts with NOD2-containing proteins that mediate oligomerization and innate immune responses (Shen et al., 2018; Jang et al., 2021). Erbin inhibits microglial pyroptosis by modulating Ca²^+^ homeostasis and mitigates neuroinflammation while enhancing cognitive function through negative regulation of the IRE1α/XBP1s-Ca^2+^ signaling pathway (Jing et al., 2022).

These findings collectively support the concept that neurons actively contribute to inflammatory regulation and provide novel experimental evidence for understanding neuron–inflammation interactions in the context of ischemic brain injury. Our findings highlight an underappreciated role of Erbin in modulating anti-inflammatory processes. Notably, Erbin markedly reduced the phosphorylation levels of ERK1/2 and p38, implicating the MAPK signaling pathway as a key mediator of its anti-inflammatory effects. Erbin has been shown to interact with activated Ras, thereby inhibiting the ERK pathway by disrupting the Ras–Raf interaction (Huang et al., 2003). Interestingly, diminished Erbin expression, accompanied by ERK hyperactivation, has been observed in a mouse model of diabetic peripheral neuropathy (Jang et al., 2021).

The present study demonstrates its inhibitory effect on the p38/MAPK pathway. Erbin upregulation in neurons enhanced neuronal survival following OGD and significantly downregulated *p*-p38 expression. These findings suggest that Erbin may confer neuroprotection against I/R injury via modulation of the p38/MAPK pathway, unveiling an underappreciated function of Erbin in MAPK signaling.

This study demonstrates that Erbin confers neuroprotection against cerebral ischemia by suppressing the MAPK pathway, a finding that may represent a microcosm of its broader role as a critical signaling hub in multiple diseases. Furthermore, the supportive evidence obtained from our cellular models strengthens the plausibility of Erbin exerting similar functions in human neurons. While interspecies differences remain a concern, this study provides solid preclinical evidence supporting Erbin as a potential therapeutic target for ischemic stroke. Recent evidence has revealed that Erbin regulates megakaryocyte function by modulating mitochondrial oxidative phosphorylation and the Erbin-endothelial cell-specific adhesion molecule interaction (Zhang et al., 2024b), improves coagulation function and organ damage in sepsis models, and coordinates cellular response networks through interactions with key signaling molecules such as Ras (Yang et al., 2025), fully demonstrating its central role in diverse physiological and pathological processes.

The neuroprotective mechanism of Erbin through inhibition of the MAPK signaling pathway offers valuable insights for its potential clinical translation to humans. Erbin is similarly expressed in the human nervous system, and its regulated MAPK pathway has been shown to play a critical role in inflammatory responses following cerebral ischemia in humans, suggesting high evolutionary conservation of this mechanism across species. However, several unresolved aspects regarding Erbin's role in inflammatory responses require further exploration. Specifically, it remains to be determined whether Erbin forms complexes with target proteins and how its binding sites are defined. Additionally, structural analyses of Erbin's leucine-rich domains and linker regions are necessary to elucidate how distinct structural elements contribute to its interactions with various signaling molecules. Future translational research may include analyzing the correlation between Erbin expression in human samples and clinical outcomes, validating its function through in vitro models, and developing targeted strategies to modulate this pathway. Future research should focus on deciphering Erbin's cell-type–specific functions, systematically mapping its interaction networks, and developing precise regulatory strategies to expand new directions for its clinical applications.

## Data Availability

The raw data supporting the conclusions of this article will be made available by the authors on a reasonable request. More data can be obtained at https://data.mendeley.com/datasets/69gznn3wx6/1.

## Synthesis

Reviewing Editor: Gergely Silasi, University of Ottawa Decisions are customarily a result of the Reviewing Editor and the peer reviewers coming together and discussing their recommendations until a consensus is reached. When revisions are invited, a fact-based synthesis statement explaining their decision and outlining what is needed to prepare a revision will be listed below. The following reviewer(s) agreed to reveal their identity: NONE.

Below you will see that R2 raised several minor points and suggestions for improving your manuscript.

Reviewer 1:

This manuscript extend the understanding of neuroprotective strategies after stroke. Accept as is.

Reviewer 2:

Authors have addressed my previous questions and concerns. Fig3-1A and B are informative.

Unfortunately, I couldn't access the additional data uploaded. Thus, I cannot judge the author's response on my western blot questions or check the additional data they provided.

Comments:

Fig. 2C. Erbin expression in different cells. In the figure legend: "C. Cellular localization of Erbin (green) in the cerebral cortex within the ischemic penumbra at 24 h after reperfusion. Brain sections from different groups were stained with Erbin..."

There is only one group. From which group are the images and where are the other groups?

What I am looking forward to seeing on this figure is Erbin expression detected by IHC after dMCAo in control vs LV-Erbin injected animals.

Lines 293-297 The following sentence is not clear. Please rephrase.

... showed that before modeling, the lentivirus-mediated overexpression of Erbin could maintain the Erbin level in the brain at a stable elevated state, while at 12 and 24 hours after ischemia reperfusion, although the Erbin level in the overexpression group also decreased to some extent, its expression level was still significantly higher than that of the corresponding control group (Fig. 3A, Table 3).

Lines 297-298 "This result indicates that the lentivirus strategy we adopted can continuously and stably maintain the high expression of Erbin at the basic and injury stages... The supplementary information (Fig. 3-1B) that you provided on LV-GFP expression after 14 days proves this point way better than expression at 24h.

Line 477 "...neurons. While interspecies differences remain a consideration, ..." Replace consideration by "concern".

Lines 479 and 480 "Erbin regulates megakaryocytes function(Zhang et al. 2024b) improves coagulation function..." For clarity, write out what is the specific function Erbin regulates.

Figure 3A, labels are wrong: Erbin (upper protein band) is 55kDa while p-Tubulin 180kDa.

Methods: Please also add the promoter that you used in LV vector for the mouse Erbin over expression in method section.
