# Supplementary figures and images for: Erbin Confers Neuroprotection against Cerebral Ischemia–Reperfusion Injury in Mice via MAPK Pathway Inhibition
Source: eNeuro. 2026 May 12;13(5):ENEURO.0089-25.2026. doi: 10.1523/ENEURO.0089-25.2026 (PMC13183369; doi:10.1523/ENEURO.0089-25.2026)

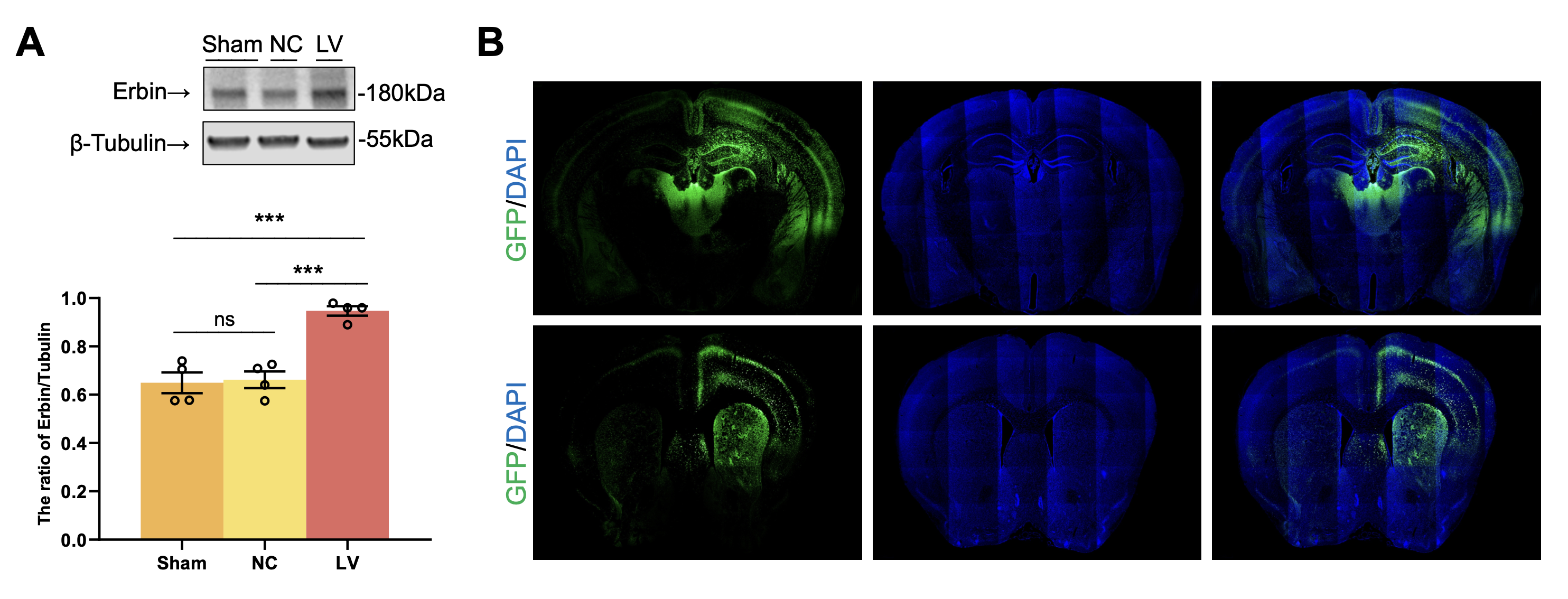

Supplement: Figure 3-1 — A. Lentivirus-mediated Erbin overexpression in the mouse brain. Western blot analysis (top) and corresponding densitometric quantification (bottom) of ipsilateral brain collected two weeks after intracerebroventricular injection. Mice were treated with either an Erbin-expressing lentivirus (LV), a control empty lentivirus (NC), or were left as Sham. The control empty lentivirus did not alter endogenous Erbin levels. Data were normalized to β-Tubulin as a loading control and are presented as mean ± SEM (n=4 per group). ***P < 0.001 versus the LV group. B. In vivo distribution of the Erbin-overexpressing lentivirus. Immunofluorescence image of a coronal brain section prepared two weeks after intracranial injection of the GFP-tagged lentivirus into the right lateral ventricle. The image shows robust viral transduction, as indicated by GFP signal (green), with prominent expression observed in the ipsilateral cortex, striatum, and hippocampus. This result serves as a visual demonstration of viral expression and distribution under basal conditions prior to injury. Download Figure 3-1, TIF file. [file eneuro-13-ENEURO.0089-25.2026-s003.tif]
