# Supplementary material for: Erbin Confers Neuroprotection against Cerebral Ischemia–Reperfusion Injury in Mice via MAPK Pathway Inhibition
Source: eNeuro. 2026 May 12;13(5):ENEURO.0089-25.2026. doi: 10.1523/ENEURO.0089-25.2026 (PMC13183369; doi:10.1523/ENEURO.0089-25.2026)
Supplement: Figure 4-1 — Statistics associated with Figure 4. Data are presented as mean ± SEM for four experimental groups: Sham, LV, I/R+NC, and I/R+LV. Behavioral assessments included the cylinder test (forelimb placing and asymmetry), grip strength, and the pole test (score, T-turn time, and descent time). Two-way ANOVA was applied as indicated. Statistical comparisons between groups are shown in the table, with significance denoted by P values or “ns” (not significant). Download Figure 4-1, DOC file. [file eneuro-13-ENEURO.0089-25.2026-s004.doc]

Figure 4-1. Statistics associated with Figure 4

| Category | Measure | Statistical test | Sham | LV | I/R+NC | I/R+LV | F test  (lentiviral vector) | F test p value(lentiviral vector) | Sham vs. LV | Sham vs. I/R+NC | Sham vs. I/R+LV | I/R+NC vs. I/R+LV |
| --- | --- | --- | --- | --- | --- | --- | --- | --- | --- | --- | --- | --- |
| Behavioral Tests | Cylinder Test forelimb placing 3d | Two-way ANOVA | 0.2933  ±  0.8383 | 0.6793  ±  0.9953 | 36.93  ±  3.729 | 24.71  ±  3.641 | F (1, 32) = 3.923 | P=0.0563 | ns | <0.0001 | <0.0001 | 0.0259 |
|  | Cylinder Test forelimb placing 7d | Two-way ANOVA | 0.1630  ±  0.6569 | -0.007259  ±  0.8825 | 29.90  ±  2.921 | 18.22  ±  3.407 | F (1, 32) = 5.315 | P=0.0278 | ns | <0.0001 | 0.0001 | 0.0106 |
|  | Cylinder Test forelimb placing 14d | Two-way ANOVA | -0.06989  ±  1.124 | -0.1281  ±  1.237 | 24.28  ±  2.823 | 14.46  ±  1.943 | F (1, 32) = 5.715 | P=0.0229 | ns | <0.0001 | 0.0001 | 0.0070 |
|  | Cylinder Test asymmetry 3d | Two-way ANOVA | -0.06250  ±  0.1990 | -0.1250  ±  0.2266 | 14.90  ±  1.157 | 0.6745  ±  10.30 | F (1, 32) = 5.569 | P=0.0245 | ns | <0.0001 | <0.0001 | 0.0085 |
|  | Cylinder Test asymmetry 7d | Two-way ANOVA | 0.000  ±  0.1890 | 0.000  ±  0.2673 | 13.15  ±  1.272 | 8.750  ±  1.121 | F (1, 32) = 5.202 | P=0.0294 | ns | <0.0001 | <0.0001 | 0.0103 |
|  | Cylinder Test asymmetry 14d | Two-way ANOVA | -0.1250  ±  0.2266 | 0.000  ±  0.2673 | 10.50  ±  0.8165 | 5.950  ±  0.8415 | F (1, 32) = 10.66 | P=0.0026 | ns | <0.0001 | <0.0001 | 0.0001 |
|  | Grip Strength 3d | Two-way ANOVA | 244.6  ±  3.056 | 245.7  ±  3.345 | 140.7  ±  2.939 | 152.0  ±  2.384 | F (1, 32) = 4.477 | P=0.0422 | ns | <0.0001 | <0.0001 | 0.0338 |
|  | Grip Strength 7d | Two-way ANOVA | 246.9  ±  2.733 | 246.6  ±  3.241 | 168.9  ±  1.808 | 186.6  ±  2.983 | F (1, 32) = 10.22 | P=0.0031 | ns | <0.0001 | <0.0001 | 0.0002 |
|  | Grip Strength 14d | Two-way ANOVA | 246.7  ±  3.043 | 244.7  ±  2.383 | 191.2  ±  2.593 | 208.8  ±  3.466 | F (1, 32) = 6.758 | P=0.0140 | ns | <0.0001 | <0.0001 | 0.0006 |
|  | Pole test score | Two-way ANOVA | 0.2083  ±  0.08768 | 0.2500  ±  0.1045 | 1.800  ±  0.2233 | 1.067  ±  0.2475 | F (1, 32) = 3.081 | P=0.0888 | ns | <0.0001 | 0.0209 | 0.0415 |
|  | Pole test T-turn 3d | Two-way ANOVA | 1.542  ±  0.1250 | 1.625  ±  0.1327 | 5.117  ±  0.8861 | 2.667  ±  0.4157 | F (1, 32) = 4.522 | P=0.0413 | ns | <0.0001 | ns | 0.0120 |
|  | Pole test T-turn 7d | Two-way ANOVA | 1.583  ±  0.1637 | 1.250  ±  0.1220 | 4.067  ±  0.8207 | 2.000  ±  0.4417 | F (1, 32) = 5.087 | P=0.0311 | ns | 0.0121 | ns | 0.0312 |
|  | Pole test T-turn 14d | Two-way ANOVA | 1.250  ±  0.1373 | 1.292  ±  0.1718 | 2.800  ±  0.4693 | 1.633  ±  0.2247 | F (1, 32) = 3.324 | P=0.0776 | ns | 0.0063 | ns | 0.0374 |
|  | Pole test time 3d | Two-way ANOVA | 5.958  ±  0.4055 | 6.167  ±  0.5040 | 52.73  ±  5.636 | 28.80  ±  7.381 | F (1, 32) = 5.141 | P=0.0303 | ns | <0.0001 | 0.0206 | 0.0087 |
|  | Pole test time 7d | Two-way ANOVA | 6.292  ±  0.3803 | 5.958  ±  0.4474 | 27.20  ±  2.659 | 18.00  ±  2.542 | F (1, 32) = 5.223 | P=0.0291 | ns | <0.0001 | 0.0021 | 0.0119 |
|  | Pole test time 14d | Two-way ANOVA | 6.250  ±  0.4910 | 6.208  ±  0.4128 | 12.35  ±  1.228 | 8.900  ±  1.053 | F (1, 32) = 3.354 | P=0.0764 | ns | 0.0004 | ns | 0.0495 |
